# The Influence of 2-Year Changes in Physical Activity, Maturation, and Nutrition on Adiposity in Adolescent Youth

**DOI:** 10.1371/journal.pone.0162395

**Published:** 2016-09-08

**Authors:** Javier Benítez-Porres, José Ramón Alvero-Cruz, Margarita Carrillo de Albornoz, Lorena Correas-Gómez, Jesús Barrera-Expósito, Manuel Dorado-Guzmán, Justin B. Moore, Elvis A. Carnero

**Affiliations:** 1 Biodynamic and Body Composition Laboratory, University of Málaga, Málaga, Spain; 2 Exercise Physiology Laboratory, University of Málaga, Málaga, Spain; 3 Department of Family & Community Medicine, Wake Forest School of Medicine, Winston-Salem, North Carolina, United States of America; 4 Translational Research Institute for Metabolism and Diabetes, Florida Hospital, Orlando, Florida, United States of America; Children's National Health System, UNITED STATES

## Abstract

The aim of this longitudinal study was to explore temporal patterns of physical activity (PA) and adiposity in Spanish adolescents. Eighty healthy adolescents were followed over a 2-year period (42 girls and 38 boys). A PA score was estimated using the Physical Activity Questionnaire for Adolescents (PAQ-A). Adiposity was assessed by anthropometric measurements; body mass index (BMI) and fat mass percent (FMP) were calculated using standard equations. Sexual maturity was estimated by percentage of predicted adult stature. Dietary intake was assessed by a self-administered food-frequency questionnaire. Three assessments were performed: September 2011 (S1), 2012 (S2), and 2013 (S3). A repeated-measures ANOVA was conducted to examine temporal changes in PA and FMP and sex change in maturation categories (two factor mixed-design, 2x2x3). A stepwise linear regression was conducted in order to estimate the predictors of FMP change. Significant changes for FMP were found between S1, S2, and S3 (23.41±8.24 vs. 21.89±7.82 vs. 22.05±8.06, p<0.05; respectively); a significant interaction of FMP with sex was observed (F = 4.387, p<0.05 for S2-S3), but not for maturation. PA at S2 was significantly higher than S3 (2.58±0.72 vs. 2.29±0.73, p<0.001). An interaction between PA change and sex was statically significant (F = 4.889, p<0.05 for S2-S3). A reduction in PA was observed after the S2 period without changes in adiposity. In contrast, a significant reduction in FMP was seen between S1 and S2, while PA did not significantly change. There were no significant differences for nutritional variables between S1 and S3, and nutrition was not a determinant of the changes in PA or FMP. Our results suggest that body composition changes observed during adolescence are not driven by changes in PA. Moreover, the interaction analysis suggests that PA behavior is affected by sex, but is not modified by maturation.

## Introduction

The prevalence of adolescent obesity has increased globally in recent decades [1, 2]. However, as changes in adiposity during adolescence are influenced by a myriad of factors (eg, age, maturation, sex, diet), the unique contribution of physical activity (PA) to inappropriate gains in adiposity during this maturational stage is unknown. It has been previously reported that PA during adolescence can result in positive effects on adult health [3] and track from adolescence to adulthood [4], which suggests that public health efforts to promote PA must start early in life [5]. However, these efforts require knowledge of the biological determinants of PA change in adolescence, and little is known about the relationship between the maturation timing of boys and girls and PA changes in adolescence.

Longitudinal observational studies aid researchers in understanding determinants of PA and other mediators of adiposity [6]. An advantage of prospective longitudinal designs is that they can address reverse causality. The interaction between PA and maturation has been studied in many contexts, namely: childhood-adolescence [7], adolescence [8–11], and childhood-adulthood [4], most of them including body composition variables [12, 13]. However, the tracking of PA and body composition, while considering the influence of nutrition and maturational status during adolescence, has been rarely studied utilizing a longitudinal approach. A longitudinal approach that considers a broader set of biological and behavioural variables will be useful in guiding future public health PA policies and interventions for youth.

The literature supports the contention that PA declines during adolescence [14–20] and that boys are more active than girls during the circumpubertal years whether PA is measured via self-report [21] or objectively [22]. Although it is acknowledged that many intra-personal, inter-personal, and environmental factors exert an important role in PA changes as youth transition into adolescence [23], preliminary research suggests that the adolescent declines in PA may be closely associated with biological age [15, 24, 25]. However, the results are inconsistent across studies [26]. While Cumming et al. [25] concluded that sex-related differences in biological maturity contribute to sex-related differences in PA during adolescence, Fawkner et al., in a longitudinal study, reported that neither maturation nor absolute changes in physical size appear to directly influence changes in PA in adolescent girls [9]. Therefore, the influence of sex, age, and maturation on changes in PA during adolescence remains equivocal.

In light of the inconclusive evidence, it is important to further explore the relationship between PA, adiposity, nutrition, and maturation during adolescence period, which has not been extensively studied. The aim of this study was to longitudinally explore PA and adiposity changes in students during adolescence. Additionally, we hoped to analyze the effects of sex, maturation, and nutritional variables on changes in PA and body composition. Based on the previous literature in children and adolescents, we hypothesized that, a) boys will be more physically active than girls at all waves of observation, but self-reported PA will decline for both sexes during adolescence, b) sex and maturation will moderate changes in body composition and self-reported PA.

## Materials & Methods

### Sample

An invitation to participate in the study was sent to all parents who had adolescent youth enrolled in schools of secondary education in Málaga and Ronda (Spain) during the beginning of the academic year in 2011. All eligible students received an information sheet and written informed consent form for parents to review, and were asked to return the forms to their school. Parents of one hundred and twenty-three potential participants who received detailed information about the aims and procedures of the study provided written informed consent. A final analytical sample of 80 presumably healthy adolescents provided longitudinal data (42 girls and 38 boys) after excluding those youth (n = 43) with incomplete data at one of the three observational periods. There were no differences in age or body mass index (BMI) between the excluded participants and those in the final analyzed sample.

The research protocol was reviewed and approved by the Ethics Committee of the Sports Medicine School, at the Faculty of Medicine (Málaga, Spain). The study was developed following the ethical guidelines of the Declaration of Helsinki-Seoul, last modified in 2008.

### Measures

#### Body composition

Participants’ heights were assessed with socks and shoes removed using a stadiometer (SECA Leicester, Birmingham, UK). A Tanita UM-050 digital weighing scale (Tanita UK Ltd, Yiewsley, Middlesex, UK) was used to assess body mass. Body mass index (BMI; weight/height; kg/m^2^) was then calculated.

Anthropometric measurements, including skinfolds (triceps, subscapular, abdominal, thigh, and calf), height and body mass, were performed from the certified personnel by International Society for the Advancement of Kinanthropometry (ISAK), according to the ISAK standards for anthropometric assessment [27]. Fat mass percent (FMP) was calculated using Slaughter´s equation [28].

#### Physical activity assessment

PA was assessed using the Physical Activity Questionnaire for Adolescents (PAQ-A) [29]. The PAQ-A is a 9-item, 7-day PA recall designed for use with older adolescents in a field-based setting. A ninth item not used in calculation of the activity score asks adolescents if they were sick or otherwise prevented from engaging in regular PA.

The PAQ-A is designed to be administered once and asks adolescents to recall their participation in activities over the last 7 days to compute an activity score, but it is not intended to estimate metabolic-equivalent expenditure. The PAQ-A has previously acceptable reliability and convergent validity [30], and is an appropriate instrument for measuring PA in Spanish adolescents [31]. The mean of all items is used to indicate the level of PA. A high score indicates higher levels of PA.

#### Sexual maturity status

Sexual maturity was assessed using the criterion of predicted percentage of maturity (adult stature). Briefly, the percentage of predicted mature (adult) height attained during measurement was used as an objective indicator of biological maturation. The method assumes that among adolescents of the same chronological age, the child that is closer to his or her predicted mature height is more advanced in biological maturity [32]. For example, a girl who has attained 95% of her predicted adult height at 12 years is considered biologically more mature than a girl of the same age who has attained 85% of her predicted adult height.

The Khamis-Roche method [33] was used to predict the mature height from current age, height and weight of the participant, and mid-parent height (average height of biological parents). Biological parents of the students reported their heights. Although this method was originally derived from data collected in the United States, European boys and girls aged 9–15 years present very similar growth trajectories and mean values for height and weight to those observed in American youth [34, 35]. The median error bound (median absolute deviation) between actual and predicted mature height from 4 to 18 years of age are 2.2 and 5.3 cm in males, and 1.7 and 4.3 cm in females, respectively [33]. Intraclass correlation coefficients between the predicted adult stature at 11 and 13 years of age were 0.95 for males and 0.89 for females.

Percentage of mature stature has been used as an index of biological maturation in a study involving US and European youth [36, 37] and has been validated against established indicators of maturity (skeletal age) in US youth [38]. It has also demonstrated an acceptable concurrent validity in European adolescents [39].

Percentages of predicted mature height were expressed as z-scores relative to age-specific means and standard deviations for percentage of mature height attained. Z-scores were used to estimate maturity status: on time (z-score between -1.0 and +1.0); late (z-score < -1.0); and early (z-score > +1.0). Relative skeletal age, the difference between skeletal age and chronological age, was used as the criterion. “On time” was defined as a skeletal age within 1.0 year of chronological age. “Late maturing” was defined as a skeletal age behind chronological age by more than 1.0 year. “Early maturing” was defined as a skeletal age in advance of chronological age by more than 1.0 year [32].

#### Food-frequency questionnaire

Dietary intake was assessed by a self-administered, semi-quantitative food-frequency questionnaire (FFQ). The FFQ was an electronic version based on a questionnaire designed for use in Spain [40]. The questionnaire consisted of 290 specific foods or food groups (including 15 fruit items and 28 vegetable and legume items) with 9 response options ranging from “never” to “6 or more times per day” for the frequency of consumption of specified serving sizes. Questions on cooking methods, specific types of fats, oils, margarines, breakfast cereals, takeaway foods, and self-prescribed nutritional supplements were also included on the questionnaire. Participants were asked to recall their frequency of consumption for common serving sizes of food per month, week, or day over the preceding six months. Afterwards, a custom Excel-based macro was used to calculate calories and macronutrients composition from each food multiplied by number of serving sizes and extrapolated for day. Dietary intake assessment was only performed at S1 and S3.

### Procedure

**Fig 1:**
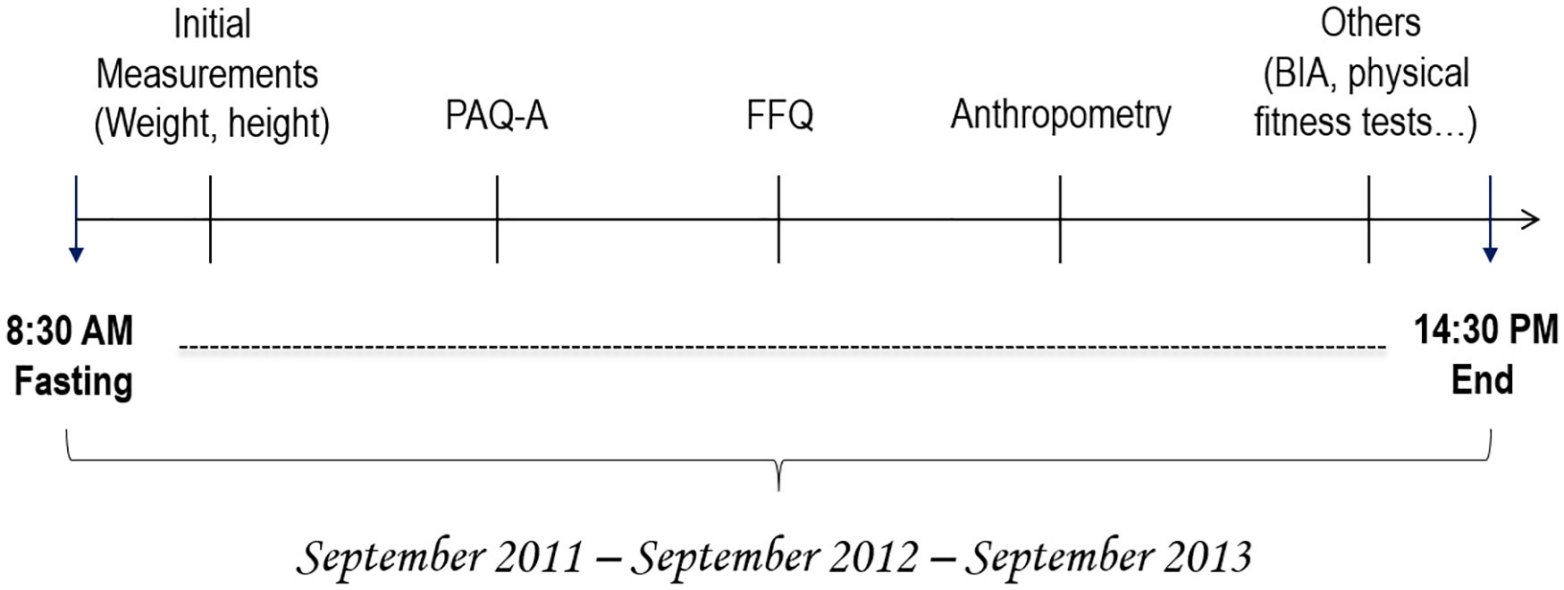
Assessment procedure.

All procedures described below were performed consistently on a single day at three waves data collection that occurred in September 2011, 2012, and 2013 (S1, S2, and S3, respectively). The procedures for data collection were executed as follows: Adolescents, who returned completed consent forms, reported to the sport facility of the school at the usual opening time (8:30 AM) in a fasting condition. A general overview of the study was provided orally by the leader of the research team. Following the introductory session, weight and height were measured, followed by a full explanation of the questionnaires to be administered. The administration of the PAQ-A and FFQ questionnaires followed. Upon completion of the questionnaires, participants were sent to the body composition assessment station, where measurements were carried out by research assistants using standard protocols. Specifically, same-sex pairs were taken into a private room where anthropometric measurements were taken by a research assistant and a secondary observer (eg, two researchers were always present in the room). At waves S2 and S3, approximately 12 months and 24 months later, data were collected using identical procedures described above (Fig 1).

### Statistical analysis

Descriptive characteristics of participants were calculated as means and standard deviations (SD). Spearman rank correlation coefficient was used to explore associations between variables. A repeated measures ANOVA (two-factor mixed model 2x3x3) was carried out among 3 time points for PA, BMI, and FMP, and compared by maturation level and sex.

Differences between baseline and second year were calculated for FMP, PA, and nutrition and maturation level. A general linear model was used to estimate predictors of FMP changes, where PA, nutrition, sex, and change in maturation level (as change of early (C0) or late state (C2) to on time and no change (C1)) were selected as independent variables. Interactions among sex, PA, nutrition, and maturation level were explored.

Regarding statistical power analysis, if we assume an alpha value of 0.05 for a multiple linear regression with 4 predictors (PA, nutrition, maturation, and sex), our final sample size of 80 adolescents will permit us to confirm our statistical analysis with 78.5% of power. The analyses were performed using SPSS 22.0 (SPSS Inc. Chicago, Illinois) and the level of significance was set at p<0.05.

## Results

Descriptive statistics at baseline (S1), follow-up years 1 (S2) and 2 (S3) for chronological age, body composition, biological maturity, PA, and nutrition are summarized, by sex, in Table 1. Characteristics of the participants are reported as mean and standard deviations. Significant differences between boys and girls were found for weight, height, FMP, predicted adult stature, and PAQ-A score.

**Table 1:** Comparison of Characteristics of Participants at Baseline and Years 1 and 2 by Sex (n = 80).

	S1		S2		S3		S1-S2	S2-S3
	Girls	Boys	Girls	Boys	Girls	Boys	*sig*.	*sig*.
**Age (years)**	14.5±1.8	14.6±2.6	14.8±1.7	15.1±2.4	16.2±1.6	16.1±2.3	†††	†††
**Weight (Kg)**	52.1±12.7	55.4±12.2	52.4±12.1*	59.6±12.8	54±11.3**	60.8±10.1	†††	
**Height (cm)**	157.5±7.1	162.4±13.7	159.5±7.0**	166.2±11.7	161.1±6.1***	168.7±10.7	†††	†††
								§§
**BMI (Kg/m2)**	20.9±4.5	20.8±3.0	20.4±3.7	21.4±3.1	20.8±4.0	21.3±2.5	†††	
**FMP (%)**	25.6±7.0*	21.0±8.9	24.6±6.8**	19.5±8.0	25.5±6.8***	18.2±7.6		§§
**Predicted adult stature (cm)**	162.5±5.0***	172.9±6.9	164.3±4.5***	173.8±5.6	163.9±5.2***	173.5±5.4		
**Predicted adult stature (%)**	97.1±4.4*	93.5±9,3	97.2±4.1	95.5±8.4	98.6±2.3	97±6.8	†††	†††
							§§§	§§§
**PA Total Score (PAQ-A)**	2.3±0.9**	2.8±0.7	2.2±0.6***	3.0±0.6	2.1±0.7**	2.6±0.6		†††
								§§§
**FFQ variables**								
*Carbohydrate (%)*	44.7±6.8	46.6±5.8	-	-	45.8±7.2	46.5±6.7		
*Protein (%)*	15.4±3.1	14.3±2.4	-	-	15.7±4.2	15.1±3.9		
*Fat (%)*	39.7±6.7	39±5.6	-	-	38.4±5.3	38.1±6.0		
*Energy (kcal/day)*	3186±1540	3427±1757	-	-	2197±998	2565±1501	‡‡‡	

S1, S2, S3 (September 2011, 2012 and 2013 respectively); BMI, Body mass index; FMP, Fat mass percent; PA, Physical activity; FFQ, Food frequency questionnaire.

* p<0.05;

** p<0.01,

*** p<0.001; independent sample t test between boys and girls.

^†††^ p<0.001; repeated measures among three moments (time factor).

^§§^ p<0.01;

^§§§^ p<0.001; interaction between time and sex.

^‡‡‡^ p<0.001; repeated measures between S1 and S3.

### Associations of body composition and physical activity

Correlations by 3 assessment groups were variable by wave: In S1, FMP was positively associated with protein intake, predicted adult height (cm), and BMI (Rho = 0.24, p<0.05; Rho = 0.27, p<0.05; Rho = 0.69, p<0.001; respectively), and inversely with predicted adult stature (%) (Rho = -0.39, p<0.01). Meanwhile, there were significant associations between the total PAQ-A score and age and predicted adult stature (%) (Rho = -0.39; Rho = -0.38; all p<0.001; respectively). These associations were stronger in girls, in which maturation level was positively associated with FMP (Rho = 0.50; p<0.001) and negatively with PA (Rho = -0.44; p<0.05).

These correlations were maintained partially at S3 but not at S2. At S3there were significant associations between PAQ-A total score and age (Rho = -0.34, p<0.05), and FMP with predicted adult stature (Rho = -0.35; p<0.05).

### Changes in body composition and physical activity and interactions with sex

**Fig 2:**
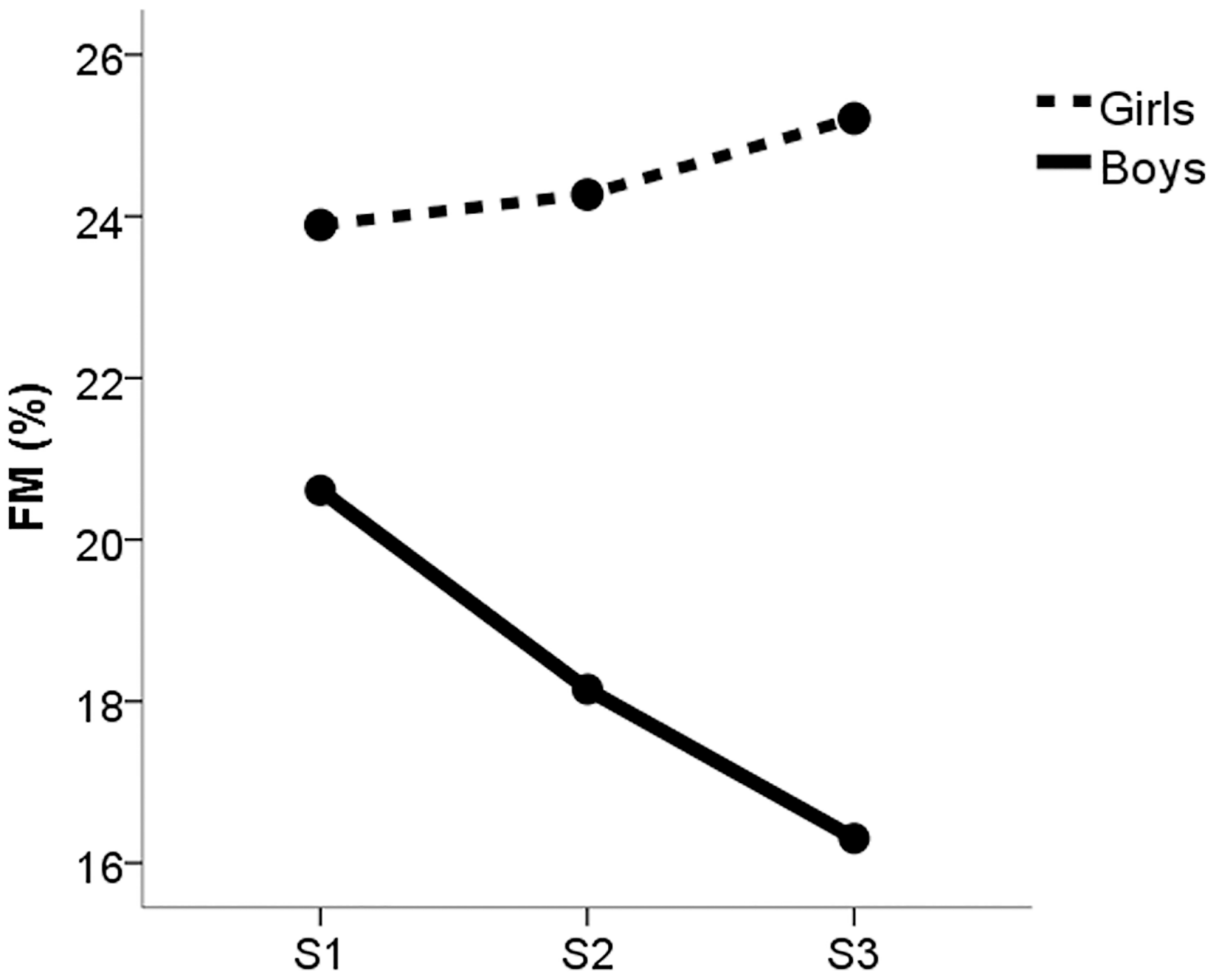
Changes in fat mass percentage at years 1 (S2) and 2 (S3) compared with baseline (S1).

**Fig 3:**
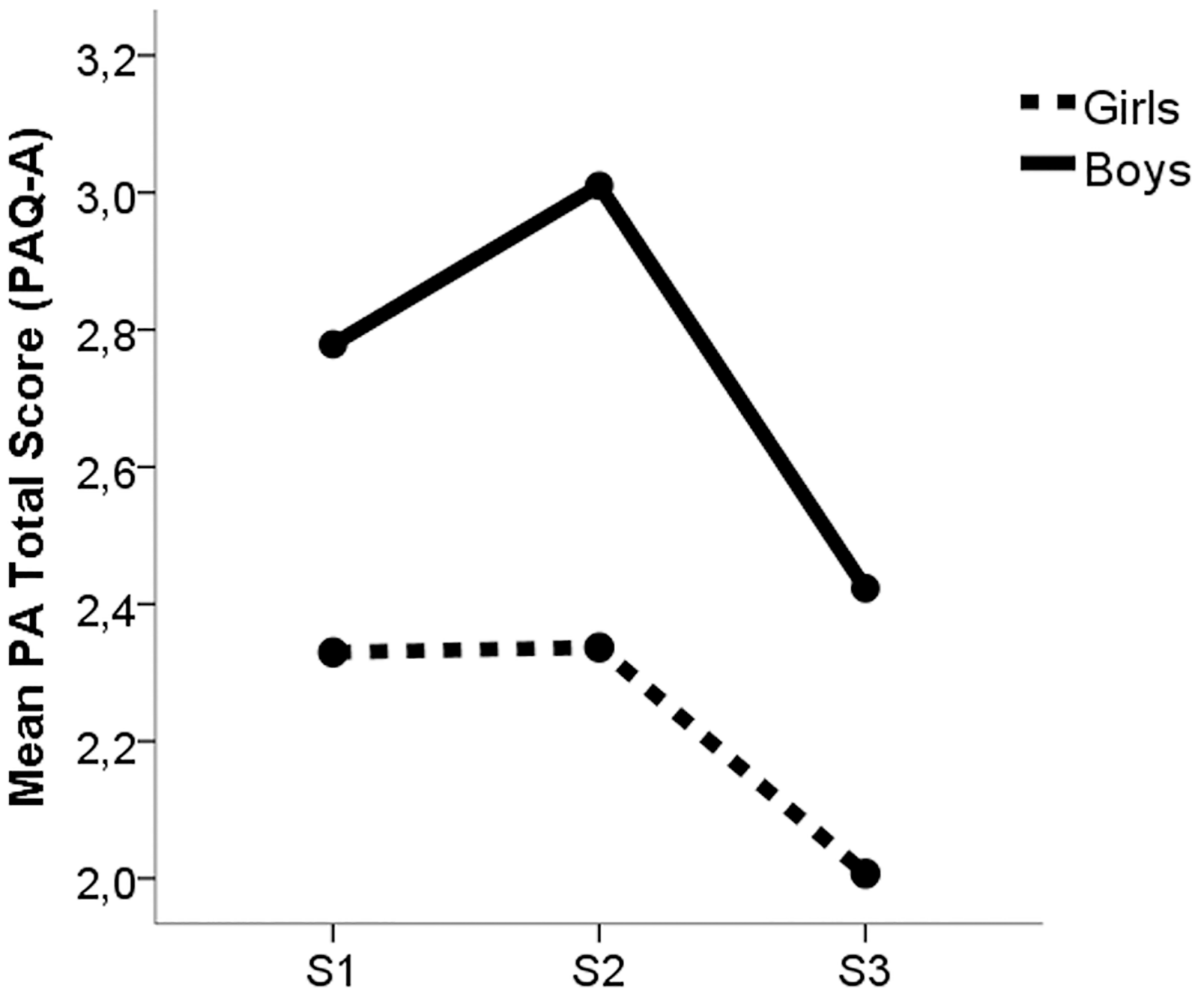
Changes in physical activity at years 1 (S2) and 2 (S3) compared with baseline (S1).

Figs 2 and 3 show changes in FMP and PA at waves S2 and S3 compared with baseline (S1). Significant differences for FMP were found among S1, S2, and S3 (23.41±8.24 vs. 21.89±7.82 vs. 22.05±8.06, p<0.05; respectively); a significant interaction with sex was observed for changes at S2 and S3 (F = 4.387, p<0.05), indicating that reductions in FMP were significantly greater in boys than girls (Table 1 and Fig 2); no interaction was found for maturation. Regarding PA, S2 was significantly higher than S3 (2.58±0.72 vs. 2.29±0.73, p<0.001). An interaction between PA and sex was statically significant (F = 4.889, p<0.05), which indicated boys displayed a larger PAQ-A total score at S2 than girls (3.01±0.57 vs. 2.32±0.63), but displayed significantly greater reductions in total PAQ-A score at S3 than girls (Table 1 and Fig 3). There were no significant differences for nutritional variables between S1 and S3.

### Maturity classification

Table 2 shows dependent and independent variables by maturity status classification. There were significant differences among maturity status (late, on time, and early) for FMP between boys and girls. In S3, late girls had 8.10±2.55% more FM than boys, and 9.12±2.78% more FM than on time girls after adjusting for nutritional variables and PA.

**Table 2:** Dependents and Independents Variables by Maturation Level.

		S1	S2	S3
Age (years)	Late	15.6±0.8 (7)	15.9±0.9 (6)	17.1±0.7 (18)
	On time	14.5±2.4 (44)	14.7±2.2 (38)	15.5±1.9 (33)
	Early	13.9±2.5 (16)	14.7±3.0 (10)	16.4±3.0 (16)
PA Total Score (PAQ-A)	Late	2.2±0.4 (7)	1.9±0.5 (6)	1.8±0.6 (18)
	On time	2.5±0.8 (44)	2.9±0.6 (31)	2.4±0.7 (33)
	Early	2.8±1.0 (16)	2.8±0.5 (7)	2.6±0.6 (16)
FMP (%)	Late	24.3±7.5 (7)	21.6±4.3 (6)	25.2±7.1 (18)*
	On time	23±9.0 (44)	20.9±8.6 (38)	21.4±8.8 (39)*
	Early	25.4±8.5 (16)	25.7±7.9 (10)	19.2±8.2 (16)
Energy (kcal/day)	Late	3117±1508 (7)	-	2518±1202 (18)
	On time	3124±1726 (42)	-	2333±1374 (31)
	Early	3334±1269 (16)	-	2422±1559 (16)

S1, S2, S3 (September 2011, 2012 and 2013 respectively); PA, Physical activity; FMP, Fat mass percent.

Number inside parenthesis indicates sample size for each maturation level group.

**Fig 4:**
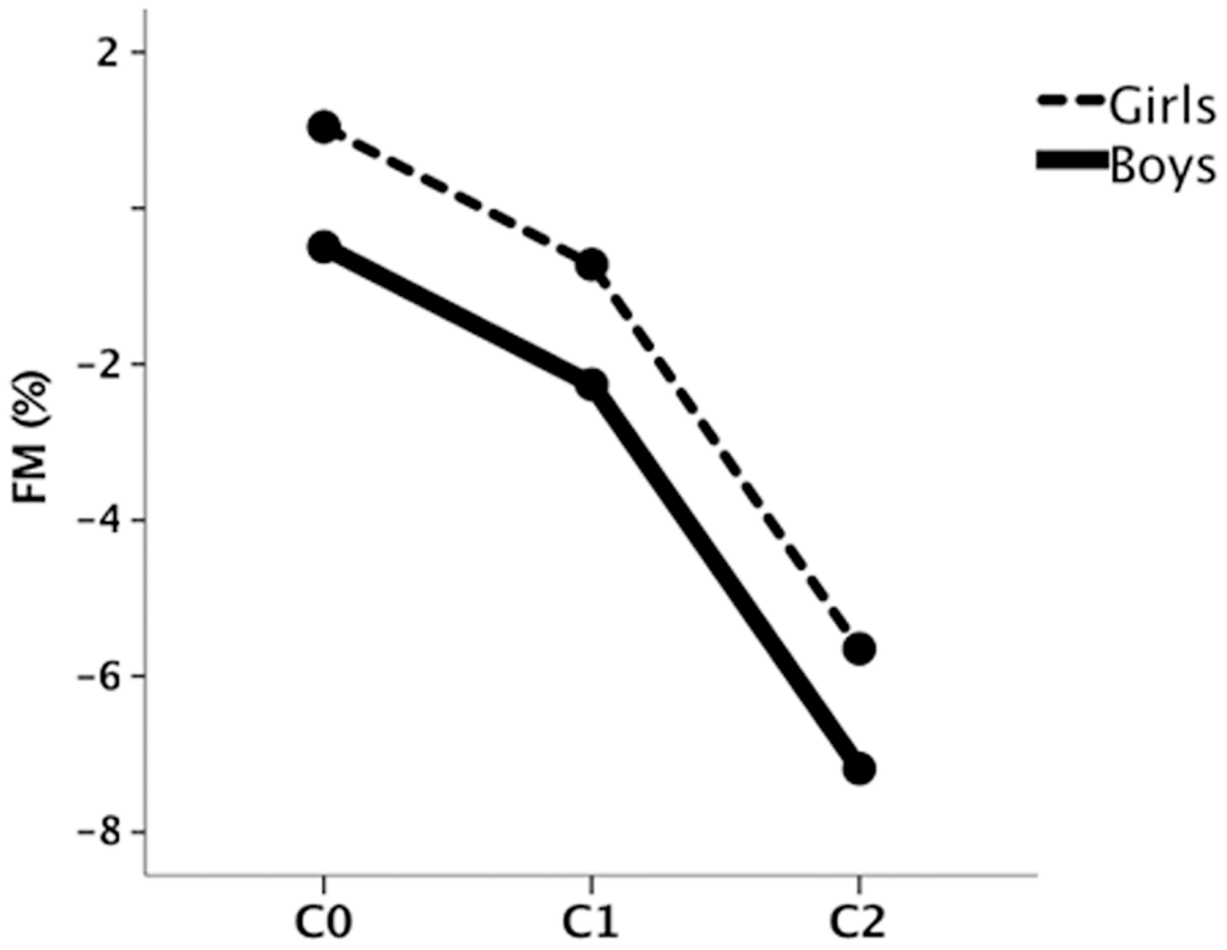
Changes in fat mass percentage after a 2-year follow-up across change in maturation status (C0, change from on time to late; C1, no change; C2, change from late/on time to on time/early) by sex. p = 0.081 and p = 0.080, for pairwise comparisons between C0-C2 and C1-C2 respectively from general lineal model analysis. p>0.05, for maturation status x sex interaction from general lineal model analysis.

A non-significant trend of FM reduction was observed across the 3 stages of change in maturation level (C0 = 0.275±2.70%; C1 = -1.490±1.10%; C2 = -6.417±2.57%; pairwise comparisons: C0—C2 = 6.69%, p = 0.081 and C1-C2 = 4.93%, p = 0.080; Fig 4).

## Discussion

The present study evaluated changes in self-reported PA and adiposity during adolescence. The main finding of this longitudinal analysis was a reduction in PA after the S2 observation period without changes in adiposity. Conversely, a reduction of FMP was only significant between S1 and S2 in boys, while PA was significantly increased. Age was negatively associated with total PAQ-A score at all times.

### Physical activity, maturation and nutrition

Our results are in accordance with findings in the literature, which suggests a decline in PA during adolescence [14–21]. Differences between boys and girls in both levels of PA and trajectories of PA were confirmed. In the present study, boys were more active than girls, while PA levels increased at S2 for boys over baseline, followed by significant declines in PA at S3 for boys. In contrast, girls declined consistently across all waves of observation. Consistent with Sallis (2000), our results provide evidence that males begin with higher levels of PA, but decline more in PA than females, specifically between S2 and S3. Davison et al. (2007) found similar decreases in PA in adolescent girls of similar chronological age. Despite these more precipitous declines, males still maintained higher levels of PA at all waves of observation.

As indicated by the analyses, maturation had an inconsequential influence on the PA behaviours at this age. Late maturing adolescents reported lower levels of PA, but this difference was not statistically significant. Early maturing adolescents may in fact be more active than their less mature peers [9], but we could not confirm maturity as an important determinant of PA in other studies [7, 26, 41]. Nutrition was also not a determinant of the changes in PA in the present sample. However, it is important to appreciate that self-report dietary assessment can introduce bias since participants providing data are aware that their dietary habits are under investigation, which may affect their reported dietary intake [42]. This is often subconscious and has been shown for adolescents, especially girls [43]. Therefore, the obtained results must be interpreted cautiously.

### Body composition, maturation and nutrition

As observed for PA changes, nutrition was not a determinant of the changes in FMP that we detected. In the present study, late and on time maturing girls had higher levels of FMP than boys. A probable explanation for the sex differences in the change in adiposity could be due to differences in lean mass between boys and girls at puberty. FMP tends to decline during male adolescence because of the rapid growth of lean mass, specifically muscle mass [32]. However, in this study the influence of maturation was similar in boys and girls since no interactions between sex, maturation, and dependent variables (PA and FMP) were found. Moreover, we observed that change in level of maturation was a determinant of change in FMP, but with a similar trend for both males and females. Those students who transitioned from a less mature state to another more mature state (C2), displayed a greater reduction in FMP (Fig 4), although this trend was similar in boys and girls.

### Strengths and limitations

The goal of this study was to explore the relationships among PA, adiposity, nutrition, and maturation, which have not been previously analysed concurrently in a longitudinal study. However, it is important to recognize a number of limitations associated with the current investigation. Firstly, the height of the parents was self-reported on a self-administered questionnaire rather than objectively measured with a stadiometer. Another potential limitation is that the method used to estimate biological maturity status was devised from data collected in the United States and further research must be required to validate the equation in Spanish youth.

An additional limitation is the use of the PAQ-A, a self-report measure, to assess PA levels. Limitations of self-report items include the tendency for people to report socially desirable responses. Despite these limitations, the PAQ-A has demonstrated to be a low cost, easy to use, and reasonably valid measure of PA behaviours that is well suited for use with Spanish adolescents [31]. Although objective measurement of PA, utilizing accelerometers, pedometers, or heart rate monitors is ideal, even these methods have their limitations (eg, cost, participant burden). Furthermore, exclusive reliance on objective measures has been criticized since accelerometers and self-report instruments measure different things [44]. Accelerometry measures body movement, while questionnaires often ask respondents to rate the activities related to the effort or frequency. Moreover, the activities reported in the first item of the PAQ-A, such as skateboarding and cycling, are difficult to be captured with accelerometers because these devices only measure acceleration from activities where center of gravity has oscillation. Additionally, questionnaires are valuable instruments to track PA in school settings, where the use of expensive and complex instruments is unviable.

## Conclusions

In summary, our findings provide longitudinal evidence that body composition and PA changes are not parallel. Also, these data suggest that changes in PA and FM are influenced more by sex than by maturation. Finally, the troubling decline in PA levels among adolescents highlights the need to develop appropriate interventions to prevent this decline.
